# Fast, Zero-Reference Low-Light Image Enhancement with Camera Response Model

**DOI:** 10.3390/s24155019

**Published:** 2024-08-02

**Authors:** Xiaofeng Wang, Liang Huang, Mingxuan Li, Chengshan Han, Xin Liu, Ting Nie

**Affiliations:** 1 Changchun Institute of Optics, Fine Mechanics and Physics, Chinese Academy of Sciences, Changchun 130033, China; wangxiaofeng201@mails.ucas.edu.cn (X.W.);; 2 University of Chinese Academy of Sciences, Beijing 100049, China

**Keywords:** low-light image enhancement, zero reference, camera response model, convolutional neural network

## Abstract

Low-light images are prevalent in intelligent monitoring and many other applications, with low brightness hindering further processing. Although low-light image enhancement can reduce the influence of such problems, current methods often involve a complex network structure or many iterations, which are not conducive to their efficiency. This paper proposes a Zero-Reference Camera Response Network using a camera response model to achieve efficient enhancement for arbitrary low-light images. A double-layer parameter-generating network with a streamlined structure is established to extract the exposure ratio K from the radiation map, which is obtained by inverting the input through a camera response function. Then, K is used as the parameter of a brightness transformation function for one transformation on the low-light image to realize enhancement. In addition, a contrast-preserving brightness loss and an edge-preserving smoothness loss are designed without the requirement for references from the dataset. Both can further retain some key information in the inputs to improve precision. The enhancement is simplified and can reach more than twice the speed of similar methods. Extensive experiments on several LLIE datasets and the DARK FACE face detection dataset fully demonstrate our method’s advantages, both subjectively and objectively.

## 1. Introduction

Due to insufficient light intensity or performance limitations of imaging equipment, low-light images inevitably exist in many machine vision applications, such as intelligent monitoring, automatic driving and mine monitoring. Especially in the field of space-based remote sensing, a large object distance makes it difficult for the sensor to receive enough energy to obtain images with high brightness. Although extending the exposure time can play a role in improvement, carriers such as satellites generally need to maintain a moving state, and it is difficult to meet the needs of both aspects at the same time. Low-light image enhancement (LLIE) helps alleviate the requirement of the movement mechanism to obtain high-quality image information. In addition to low brightness, low-light images often have problems such as poor contrast and strong noise, which hinder subsequent analysis and processing, so it is of great significance to study the enhancement of low-light images. So far, many methods have been proposed to realize LLIE. They can be broadly divided into two categories: traditional methods and deep learning-based methods.

Traditional low-light image enhancement methods are mainly based on histogram equalization [1,2,3], Retinex theory [4,5,6], wavelet transform [7], defogging or dehazing models [8,9,10] or multi-graph fusion [11]. The first two methods have been studied the most. Although the histogram equalization method has a simple principle and fast calculation, the enhancement effect is poor. Methods based on Retinex theory usually divide a low-light image into a reflection component and an illumination component based on some prior knowledge, among which the estimated reflection component is the enhancement result. However, the ideal assumption of the reflection component as the enhancement result is not always true, which may lead to degraded enhancement, with effects such as the loss of detail or color distortion. In addition, it is very difficult to find effective prior knowledge, and inaccurate prior knowledge may lead to artifacts or color deviations in the enhancement results. In addition to these mainstream research directions, LECARM [12] thoroughly studied an LLIE architecture based on the response characteristics of the camera. It analyzed the camera response model parameters and estimated the exposure ratio of each pixel using illumination estimation technology to realize the enhancement of low-light images. But, the exposure ratio estimation method used by LECARM is LIME [13] based on Retinex theory. Various parameters need to be designed manually, which is difficult to control.

Since KG Lore et al. proposed LLNet [14] in 2017, low-light image enhancement methods based on deep learning have attracted the attention of more and more researchers. Compared with traditional methods, deep learning-based solutions often have better accuracy. Such methods generally use a large set of labeled pairwise data to drive a pre-defined network to extract image features and realize enhancement. Some dehazing methods can also be used to improve brightness. SID-Net [15] integrates adversarial learning and contrastive learning into a unified net to efficiently enhance pictures. More can be found in [16]. However, in the field of LLIE, it is difficult to determine a fixed standard for the target image, and it is extremely difficult to obtain ideal paired data. Moreover, preset target images will drive the model to learn more unique features of the dataset and reduce the generalization ability. In this respect, zero-reference methods in the form of unsupervised learning can enhance low-light input images without requiring references from the dataset, which reduces the dependence on the dataset and is beneficial in expanding the adaptation scenarios of the model. Based on the idea of generation and confrontation, a zero-reference attention-guided U-Net network was designed in EnlightenGAN [17]. It uses a global discriminator, a local discriminator and self-feature preserving loss to restrain the training process. The ZeroDCE [18] proposed by Guo et al. uses a variety of images taken at different exposures to train a lightweight depth curve estimation network. A series of elaborately designed loss functions are used to realize training without references.

Although each method can achieve a certain degree of enhancement, there are still some problems. EnlightenGAN establishes the model based on a complete U-Net, which is relatively complex and is not conducive to real-time applications. The fixed-exposure loss function used by ZeroDCE ignores the brightness distribution in the original images, which will limit the contrast of the results. And, its enhancement process requires many iterations, which is contrary to high-speed processing. The subsequently proposed ZeroDCE++ [19] improves the speed by compressing the size of input images and reducing the number of convolution operations. But, it is easy to lose important information when the scale of targets of interest is small. Most other similar methods also use multiple iterations [18,19,20,21] or complicated model structures [17,22] to achieve enhancement, limiting the processing speed of the algorithm.

In order to solve the above problems, this paper proposes the Zero-Reference Camera Response Network (ZRCRN). Inspired by the CRM, the ZRCRN realizes an efficient framework. Without multiple iterations, it just uses a simple network structure to achieve high-quality enhancement, which effectively improves the processing speed. Two reference-free loss functions are also designed to restrict the training process to improve the generalization ability of the model.

The main contributions of this research can be summarized as follows:

- A fast LLIE method is proposed, namely, the ZRCRN, which establishes a double-layer parameter-generating network to automatically extract the exposure ratio of a camera response model to realize enhancement. The process is simplified, and the speed can reach more than twice that of similar methods. In addition, the ZRCRN can still obtain an accuracy comparable to SOTA methods.
- A contrast-preserving brightness loss is proposed to retain the brightness distribution in the original input and enhance the contrast of the final output. It converts the input image to a single-channel brightness diagram to avoid serious color deviations caused by subsequent operations and linearly stretches the brightness diagram to obtain the expected target. It can effectively improve the brightness and contrast without requiring references from the dataset, which can improve the generalization ability of the model.
- An edge-preserving smoothness loss is proposed to remove noise and enhance details. The variation trend in the input image pixel values is selectively promoted or suppressed to reach the desired goal. While maintaining the advantage of zero references, it can also drive the model to achieve the effect of sharpening and noise reduction, further refining its performance.

The rest of this paper is organized as follows. The related works on low-light image enhancement are reviewed in Section 2. In Section 3, we introduce the details of our method. Numerous experiments on standard datasets have been implemented to evaluate the enhancement performance and are presented in Section 4. The discussion and conclusion are finally given in the last two sections.

## 2. Related Works

### 2.1. Camera Response Model

Images are mainly obtained by digital cameras, which collect the radiation energy from the real scene through the lens and photosensitive sensors, convert the optical signals into electrical signals, and then obtain the final images through a series of digital image processing methods and store them in the memory. The whole process, from the incident radiation at the lens to the pixel values of the final images, contains many linear or nonlinear transformations, which can be represented by a camera response function (CRF). It is convenient for analysis to condense the whole process into one function.

**Figure 1:**
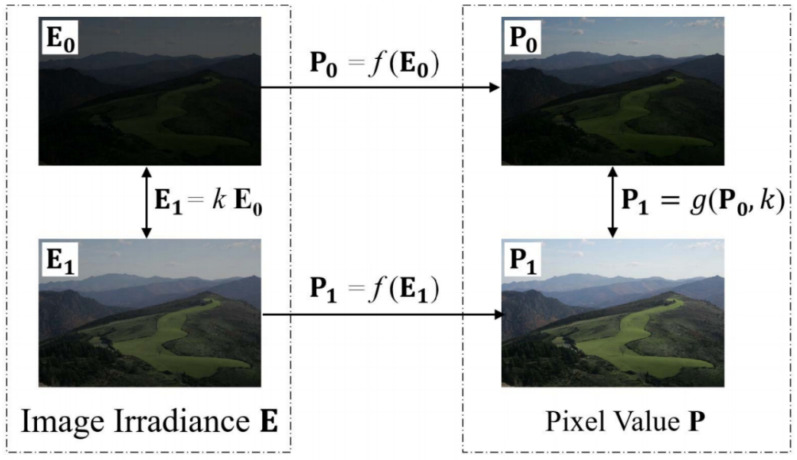
The relationship between the pixel values of the image and the incident radiation in the same scene under two different exposure conditions.

When the exposure setting of the camera changes, the radiation energy incident to the sensor will change accordingly, and these two changes should be linearly correlated. Meanwhile, the pixel values of the image output by the camera will also change, and this change can be expressed by the brightness transformation function (BTF). Denoting the CRF by f(·) and BTF by g(·), then, as shown in Figure 1, these two functions have the following relationships:

(1) $$P=f\left(E\right),\;\;E_{1}=kE_{0},\;\;\;P_{1}=g\left(P_{0},k\right),$$

in which E represents the incident radiation matrix, $E_{0}$ and $E_{1}$ are two of its index values under two different exposure conditions, $P_{0}$ and $P_{1}$ are the corresponding image pixel value matrices, and *k* is the exposure ratio.

The CRF and BTF describe the characteristics of the imaging process of a camera. Together, they form a camera response model (CRM) [12]. A relationship also exists between the CRF and BTF:

(2) $$g\left(f\left(E\right),k\right)=f\left(kE\right).$$

This equation, also known as a comparative equation, can be used to convert between the CRF and BTF [12].

The CRF can be obtained by experimentally measuring radiation intensity and image pixel values. In practice, it is generally assumed that the CRF and BTF are the same at each spatial position. DoRF [23] is a database that stores the response curves of 201 real cameras. Although different cameras should have different camera response models in theory, if the camera response model is regarded as a medium for transforming the incident radiation matrix $E_{0}$ to the perfect image $P_{1}$, and $E_{0}$ obviously has nothing to do with the camera and the goal of $P_{1}$ is to match human perception, then considering that most human perception standards are similar, the CRM should also have a general solution that can meet most requirements. The study of Yurui Ren et al. [12] proved that, for most cameras, using the average curve of the DoRF database instead of its own three-channel camera response curve for LLIE will produce only limited distortions. They also summarized a general and fast CRM, whose CRF and BTF are as shown in the following equation:

(3) $$f\left(E\right)=\left(1+a\right)\frac{E^{b}}{E^{b}+a},g\left(P,k\right)=\frac{k^{b}P\left(1+a\right)}{(k^{b}-1)P+1+a},a=0.6,b=0.9,$$

where *E* is the pixel value of the incident radiation metric, and *P* is the pixel value of the low-light image.

The CRF in this model has the form of a sigmoid function and was proposed by Gabriel Eilertsen et al. [24]. The ZRCRN establishes a network based on this model to realize LLIE.

### 2.2. Zero-Reference LLIE Loss Function

A loss function in the field of image enhancement often uses the mean absolute error or mean square error (MSE) between the enhancement result and the target image. The former is not derivable at 0. The latter is always expressed as Equation (4), with *n* denoting the count of the samples, $y_{i}$ denoting the true value of the *i*-th sample and $\hat{y_{i}}$ denoting its predicted value.

(4) $$MSE=\frac{1}{n}\sum_{i=1}^{n}{\left(\hat{y_{i}}-y_{i}\right)}^{2}.$$

Although susceptible to abnormal data, the MSE is globally derivable and is more widely applied. When no target image is provided, there are two main schemes for the establishment of the loss function: building the target image based on the original image or setting the ideal target image directly. Although the final form of the loss function is always still the mean error, the target image is no longer limited to RGB pixel values but is also characterized by a brightness map [18,19,20,21,22,25,26] (usually with a fixed value of 0.6 for improving brightness), a hue diagram [25] (H channel in the HSV space to maintain color), the difference between any two color channels [18,19,21,26,27,28] (usually with a fixed value of 0 for holding color), the difference between neighborhood brightness levels [18,19,20,21,22,25,26,27,28] (usually with a fixed value of 0 or extracted from the original image, often used for noise suppression or maintaining the brightness distribution trend) and so on. The enhancement results used to calculate the error will also be replaced by the corresponding features. In addition, there are some loss functions designed according to proprietary model structures, such as generative adversarial loss [17,29], semantic loss [21], atmospheric light loss [20], the consistency loss of neighborhood parameter values [18,19], etc. In practice, the sum of multiple loss functions is often used to achieve the purpose of brightness enhancement, contrast enhancement and noise suppression at the same time.

When a combination of multiple loss functions is used, the weight of each component should be carefully set. Otherwise, one kind of loss function can easily dominate, and other loss functions are almost ineffective. At present, most weights are determined by manual testing. There are also some semi-automatic configuration methods. For example, A. Kendall et al. [30] set the weights by analyzing the homoscedastic uncertainty of each task, while O. Sener et al. [31] solved multi-task learning as a multi-objective optimization problem and proposed a multiple-gradient descent algorithm.

The main goal of LLIE is to improve the brightness and contrast of the image and suppress noise. The commonly used brightness loss function is meant to constrain the enhancement result to a target brightness map with a fixed value at each position. Although it can improve brightness, the brightness distribution may be abnormal. And, this loss is not conducive to contrast improvement. In addition, the loss function is less able to suppress noise. The main objective of increasing the consistency of neighborhood brightness differences between the result and the original image is to maintain the brightness distribution trend. If the brightness difference tends to zero, it will lead to global smoothing, the easy loss of details and lower contrast. This paper designs two loss functions that extract key information from the original image to establish the reference target for the performance improvement of the model.

## 3. Methods

**Figure 2:**
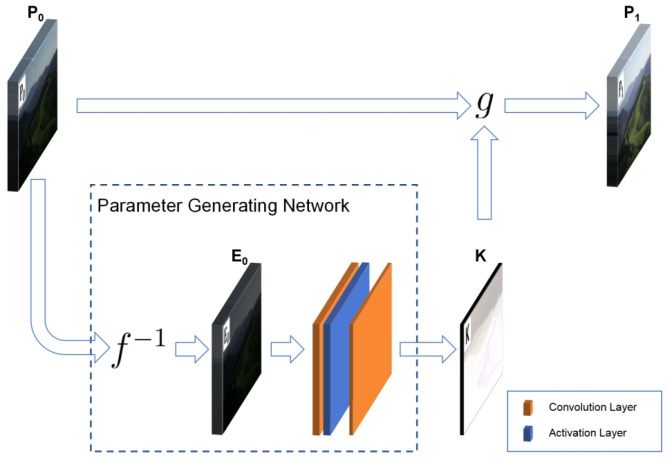
The framework of the ZRCRN. *f* is the CRF, and *g* is the BTF. The parameter-generating network includes inverse CRF transformation and convolutional model extraction with two main operations. It extracts the parameter K from the input low-light image $P_{0}$. The BTF calls K to transform $P_{0}$ to the enhancement result $P_{1}$.

The framework of the ZRCRN is shown in Figure 2. Based on the CRM, the original low-light input image $P_{0}$ is first inverted to the radiation map $E_{0}$ according to the sigmoid CRF to reduce complexity. A streamlined parameter-generating network (PGN) is then established to extract the pixel-wise exposure ratio K from the radiation map. Finally, the corresponding BTF calls K to perform one transformation on $P_{0}$ to achieve LLIE. The CRM, including the sigmoid CRF, is shown in Equation (3). The designs of the parameter-generating network and the loss functions are detailed below.

### 3.1. Parameter-Generating Network

The image quality in low-light scenarios can be improved by adjusting the camera exposure parameters. Extending the exposure time or increasing the aperture size can increase both the brightness and contrast of the final image. At the same time, according to the CRM, the adjustment of exposure parameters can be represented by the parameter *k*. As long as an appropriate *k* can be obtained, LLIE can be realized by the CRF or BTF.

In LLIE tasks, *k* represents the exposure ratio between the low-light image and the normal-light image. The ideal normal-light image should have a fixed average brightness, and the camera needs to be configured to different exposures for different scenes to obtain this target value. In practice, the scene and exposure of the low-light image are fixed, so the exposure ratio should be a function of the low-light input image. In addition, considering that low-light images often have the problem of uneven illumination, which also needs to be tackled in the enhancement, the optimal exposure ratio should vary at different coordinates. Accordingly, the exposure ratio should be a matrix matching $P_{0}$ pixel by pixel, and the objective function φ should meet the following relationship:

(5) $$K=\varphi \left(P_{0}\right),$$

in which K is the exposure ratio matrix. Although K can be obtained from the features extracted from $P_{0}$ directly, a deeper and more complex model may be needed to achieve a good fit since $P_{0}$ is obtained by the camera through a CRF transformation, and the CRF is a collection of many transformations. In addition, the main function of K is to raise the environmental radiation to the level of the normal illumination scene, which is independent of the complex CRF transformation inside the camera. Therefore, the exposure ratio parameter K required by the enhancement can be obtained by the following process: first, inversely transform $P_{0}$ by the CRF to obtain the radiation map in the low-light scene ($E_{0}$), and then extract K from $E_{0}$. This process can be expressed as the following equation:

(6) $$K=\psi \left(E_{0}\right)=\psi \left(f^{-1}P_{0}\right).$$

At this time, the required objective function (ψ) has weaker nonlinearity, which is conducive to achieving a better fit using a simpler model and improving the processing speed.

**Figure 3:**
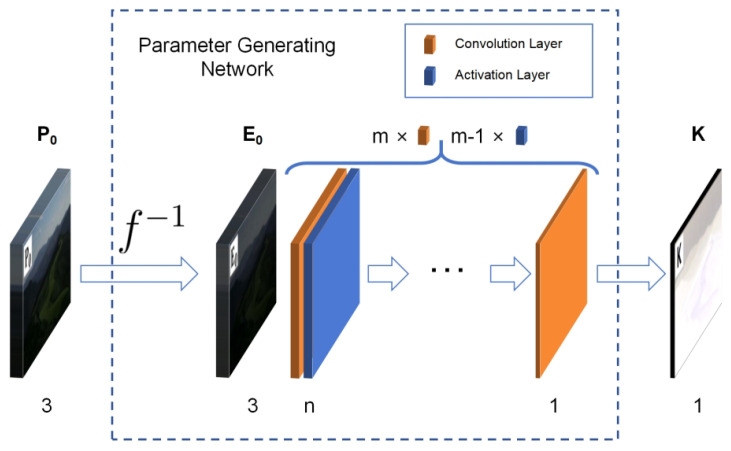
The structure of the PGN. The number at the bottom represents the number of channels of the corresponding image or convolution layer. $f^{-1}$ indicating the inverse CRF transformation. The low-light image ($P_{0}$) acquired by the camera first passes through inverse CRF transformation to obtain the low-light incident radiation map ($E_{0}$) and then passes through a series of layers to obtain the enhancement-required single-channel pixel-wise parameter diagram (K).

Convolution requires very few parameters to achieve the extraction of important information in the matrix. It has the characteristics of translation invariance and locality and is widely used in the field of image processing. The PGN is built using *m* convolution layers and m−1 activation layers, with each convolution layer followed by one activation layer, except for the last one. The kernel size of the convolution layer is 3×3. The height and width of the output image remain unchanged by padding. The activation layer uses the LeakyReLU function with a negative slope of 0.01 as the activation function. The first convolution layer has 3 channels. The last has 1 channel. All output channels of the other convolution layers are *n*. The overall parameter-generating network structure is shown in Figure 3. A larger *m* or *n* can improve the representation ability of the model. Their best values were determined to be 2 and 2 through experiments described in the next section.

### 3.2. Loss Function

The loss function plays an important role in learning-based methods. It controls the direction of the training process. With it, a model can acquire specific abilities. A loss function should be derivable to implement backpropagation. The MSE is a commonly used loss function. It measures the distance between two instances and is globally derivable. But, it needs a reference, which is not always available in a dataset. Considering that some traditional methods have unique advantages in enhancing specific properties of an image, we propose two loss functions to generate references according to the low-light inputs.

Aiming at the three main problems of low brightness, low contrast and strong noise, two loss functions are designed to constrain the training process: contrast-preserving brightness loss and edge-preserving smoothness loss. Both are essentially MSE loss functions. Some attributes of the original image are separately extracted to construct the target image with restricted enhancement. Then, the corresponding properties are calculated from the output of the network. The deviation between these two elements is minimized so as to achieve the enhancement effect.

#### 3.2.1. Contrast-Preserving Brightness Loss

The original low-light image ($P_{0}$) has three RGB channels and low pixel values in each channel. The brightness map is extracted from $P_{0}$, and linear stretching is performed to obtain the target brightness map. The specific process is as follows:

1. In order to reduce the impact on color information, the RGB channel is fused into a single channel to obtain the brightness map. The conversion formula uses the following, which is more suitable for human perception:
  (7) $$\Phi \left(x_{i}\right)=0.299\cdot r_{i}+0.587\cdot g_{i}+0.114\cdot b_{i},$$
  where $x_{i}$ represents the value of the *i*-th pixel in the image. It is a vector consisting of a red channel value ($r_{i}$), a green channel value ($g_{i}$) and a blue channel value ($b_{i}$).
2. After applying Φ(·) to each pixel of $P_{0}$, the brightness map ($E_{\mathrm{brightness}}$) is dark. Then, it is linearly stretched to enhance the brightness and contrast. The following equation is used to extend the pixel values of $E_{\mathrm{brightness}}$ linearly to the $\left[a-b\right]$ interval to obtain the expected brightness map ($E_{\mathrm{exptar}}$):
  (8) $$E_{\mathrm{exptar}}=a+\frac{b-a}{max\left(E_{\mathrm{brightness}}\right)-min\left(E_{\mathrm{brightness}}\right)}\left(E_{\mathrm{brightness}}-a\right).$$
  where max(·) and min(·) represent the maximum and minimum values of all elements in the argument matrix. Considering the purpose of increasing the brightness, *b* can be directly set as the theoretical maximum value of 1. Since the brightness distribution of the original image is concentrated close to the minimum value, the value of *a* should be more carefully determined. The larger *a* is, the smaller the width of the brightness distribution interval and the lower the contrast. If *a* is too small, the visual effect of the target image will obviously be darker, and the corresponding model will not effectively enhance the brightness of the original image. The best value of *a* was determined to be 0.2 through experiments described in the next section.

Once $E_{\mathrm{exptar}}$ is obtained, the contrast-preserving brightness loss can be calculated by the following equation:

(9) $$L_{exp}=\frac{1}{N}\sum_{i=1}^{N}{\left(\Phi \left(x_{i}\right)-y_{i}\right)}^{2},x_{i}\in P_{1},y_{i}\in E_{\mathrm{exptar}},$$

where *N* represents the total count of pixels in the image. $x_{i}$ denotes the *i*-th element (including three RGB channels) of the enhancement result ($P_{1}$). $y_{i}$ is the *i*-th element of $E_{\mathrm{exptar}}$. The brightness distribution of a low-light image is generally concentrated in a small range close to zero. Linearly stretching it to $\left[a-1\right]$ can improve the brightness and contrast. Compared with the commonly used fixed target brightness of 0.6 [18,19,21,22], the contrast-preserving brightness loss is more conducive to retaining the spatial distribution information of the brightness in the original image and improving the contrast of the enhancement results.

#### 3.2.2. Edge-Preserving Smoothness Loss

In addition to the brightness and contrast, noise is also a major problem in low-light images. Suppressing the variation trend in the input image pixel values can remove noise. The variation trends in the image pixel values are always represented as a gradient diagram. Noise appears as a large number of non-zero isolated points in the gradient diagram. To realize proper noise reduction, the gradient map and edge map are generated based on $P_{0}$, the values in the non-edge region of the gradient map are set to zero and the gradient in the edge region is properly amplified to obtain the target gradient map. Specifically, it includes the following three steps:

1. **Edge Detection.** The main basis for generating the edge diagram is the Canny algorithm [32]. Firstly, horizontal and vertical Gaussian filters are applied to $P_{0}$ to suppress the influence of noise on edge detection. The size and standard deviation of the filter inherit the default parameters of 5 and 1. Then, the gradient information is extracted. The horizontal and vertical Sobel operators of size 3×3 are used to calculate the gradient diagram in two directions of each of the three RGB channels from the filtered result. And, the amplitude of the total gradient at each position is obtained by summing the gradient amplitudes in each channel. Next, the double-threshold algorithm is used to find the positions of the strong and weak edges. Finally, the edge diagram ($E_{\mathrm{edge}}$) is obtained by judging the connectivity and removing the isolated weak edge. The edge refinement operation with non-maximum suppression from the original Canny algorithm is skipped. So, there is no need to calculate the direction of the total gradient. The main purpose is to keep the gradient edge with a larger width in the original image and make the enhancement result more natural. The two thresholds α and β of the double-threshold algorithm have a great influence on the edge detection results. Their best values, as determined by the experiments described in the next section, are 0.707 and 1.414, respectively.
2. **Gradient Extraction.** The horizontal and vertical Sobel operators are applied directly to $P_{0}$ to obtain a total of six gradient maps in three channels and two directions. Then, the total gradient amplitude (the sum of the gradient amplitudes in all channels) is calculated as the pixel value in the final gradient map ($E_{\mathrm{grad}}$). The entire gradient extraction process can be expressed as the following equation:
  (10) $$E_{\mathrm{grad}}=\Psi \left(P_{0}\right).$$
3. **Selectively Scaling.** The edge diagram ($E_{\mathrm{edge}}$) is used as a mask to multiply the gradient map ($E_{\mathrm{grad}}$) element by element, which can filter out the non-edge part of $E_{\mathrm{grad}}$. Then, the result is scaled by an amplification factor (γ) to obtain the expected target image ($E_{\mathrm{smoothtar}}$):
  (11) $$E_{\mathrm{smoothtar}}=\gamma E_{\mathrm{grad}}⨀E_{\mathrm{edge}}.$$
  where ⨀ indicates the multiplication of two metrics pixel by pixel. The gradient at the edge of the low-light image will be enhanced correspondingly after the brightness is enhanced. So, the target gradient map should also amplify this part. The best value of γ was determined to be 2 by the experiments described in the next section.

After obtaining $E_{\mathrm{smoothtar}}$, denoting the total count of elements in the image by *N*, the edge-preserving smoothness loss can be calculated by the following formula:

(12) $$L_{smooth}=\frac{1}{N}\sum_{i=1}^{N}{\left(x_{i}-y_{i}\right)}^{2},x_{i}\in \Psi \left(P_{1}\right),y_{i}\in E_{\mathrm{smoothtar}}.$$

Although each step in the calculation of the loss function is derivable, the total gradient amplitude is solved by the root operation, and the problem of dividing by 0 can easily occur. In practice, an additional minimum of 1 × 10^−8^ is added to the base number to ensure that the training process is continuous.

The gradient map of the low-light image often contains a large number of non-zero isolated points. Making them close to zero can achieve smoothness. Enlarging the gradients at the edge positions is beneficial in retaining the details of the original image. To the best of our knowledge, in zero-reference LLIE, the loss function is currently less able to suppress noise. Although some work tried to constrain the brightness difference in the neighborhood, the main objective of increasing the consistency of neighborhood brightness differences between the result and the original image [18,19] is to maintain the brightness distribution trend. If the brightness difference tends to zero [21,27,28], it will lead to global smoothing, the easy loss of details and lower contrast. The edge-preserving smoothness loss attempts to separate the edge region specifically, enhancing the variation in this area and suppressing others to build references, which can further improve precision.

#### 3.2.3. Total Loss Function

Finally, according to the multi-loss combination strategy proposed by A. Kendall et al. [30], two learnable parameters are added to combine the two loss functions together to obtain the total loss function:

(13) $$L_{total}=e^{-p}L_{exp}+p+e^{-q}L_{smooth}+q.$$

where p and q are two learnable parameters with an initial value of zero. To a certain extent, they can release us from the adjustment of weights and improve precision.

In practice, the expected target images required for each loss can be calculated according to the images in the training dataset before formal training and directly loaded during training to enhance efficiency.

## 4. Experiments and Results

### 4.1. Implementation Details

The contrast-preserving brightness loss and the edge-preserving smoothness loss leave the training of the ZRCRN without the requirement for paired data from datasets. To address the common problem of non-uniform illumination in LLIE tasks, multi-exposure image sequences from the SICE dataset [33] were selected for training, the same as ZERO_DCE [18], to give the model the ability to process input images with diverse exposure states.

The model was implemented through PyTorch and trained on a single GPU with the batch size set to 10. At initialization, the weights of all convolution layers were sampled from a Gaussian distribution with a mean of 0 and a standard deviation of 0.02, with the bias set to 0. The initial values of the two learnable parameters α and β in the loss function were also set to 0. The ADAM optimizer [34] was used to update the parameters. In addition to the learning rate, the value of each parameter was left as the default. The learning rate was configured according to the warm start + cosine annealing strategy [35]. The basic learning rate was 0.05. The learning rate increased linearly from 1% of the basic learning rate to 100% in 100 batches. The maximum learning rate of cosine annealing was also the basic value. The period was kept at 200, and the minimum learning rate was set to one-tenth of the basic learning rate. The learning rate was updated at every batch.

**Figure 4:**
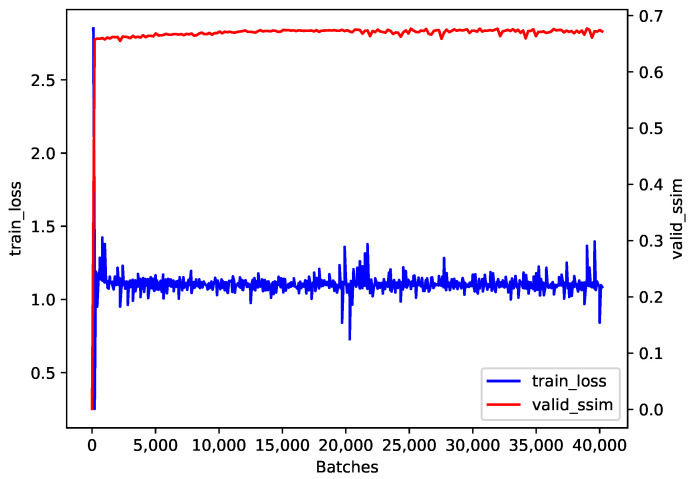
The training loss and the validation SSIM during training.

The training loss and the validation SSIM during our training process are presented in Figure 4. After the initial rapid improvement and the following slow increase, the validation SSIM tended to converge to a value near 0.7. Although the cosine annealing strategy led to some fluctuations in training loss, it converged to a value near 1.0.

### 4.2. Ablation Study

To analyze the influence of key parameters and verify the role of inverse transformation and loss functions, five sets of ablation experiments were designed for comparison. The test set used for the quantitative comparison in each experiment was the test part of the real-captured set of LOL_v2 [36]. The SSIM [37], the PSNR and the average single-image processing time (denoted as TIME, in ms, calculated by dividing the processing time of all images in the dataset by the count of images contained in the dataset) were selected for performance analysis. In all tables, the optimal results in each column are shown in bold, the second-best values are underlined and the third-best results are in italics.

#### 4.2.1. Influence of Key Parameters

**Number of Layers and Channels in PGN.** Using deeper and wider convolution structures gives the model stronger representation but leads to more computation, less efficiency and more difficulties in finding model parameters that meet the expected goals. m=1,2,3,4 and *n* = 1, 2, 4 were used to generate binary groups for the quantitative evaluation. The results are shown in Table 1. It can be seen that the scheme with *m* = 1, which obtains single-channel enhancement parameters directly from the three-channel input image, only achieved an SSIM of 0.44. It is too simple a structure to extract enough features and cannot obtain sufficient accuracy. The combination of m=n=2 achieved the highest SSIM in the test set and could process extremely fast. Structures with m>2 and n>2 failed to achieve higher accuracy with the current learning strategy. Although setting the additional convolution kernels to specific values should yield the same output result as the combination of m=n=2, a deeper and wider model structure has a larger parameter space and is more likely to fall into other local minimum points during the optimization, which leads to sub-optimal results. They also slowed down the enhancement. Considering accuracy and efficiency, a network structure with m=n=2 was chosen.

**Minimum of Linear Stretching Target Interval in Contrast-Preserving Brightness Loss.** The brightness and contrast of low-light images can be improved by linear stretching. The brightness distribution of low-light images is concentrated near zero, so the selection of the lower limit of the target interval has a great influence on the enhancement effect. A larger value will brighten the image remarkably. Values of a = 0, 0.1, 0.2, 0.3, 0.4 and 0.5, combined b with a value of 1, were taken to obtain the linear stretching target interval [a,1] for comparison. The results are shown in Table 2. When a was too large (a = 0.5) or too small (a = 0.0), the SSIM between the output of the network and the target image was degraded significantly. Although the SSIMs of the other four sets of parameters were not much different, the PSNR achieved the highest value at a = 0.2. We think that with a = 0.2, a good reference can be built for most low-light images, and a = 0.2 is chosen as the minimum of the linear stretching target interval in contrast-preserving brightness loss.

In addition, we calculated the average SSIM between all generated references under five settings and the brightness of the normal-light images in our test set. The results are shown in Table 2 under the heading “SSIM-EXPTAR2BONL”. They show a similar trend to other SSIMs, which means that our model robustly learned the ability to brighten an image, and the better the generated reference is, the more accurate the model can be. Considering that a learning process still exists between these two processes, we identified some candidate parameters based on a rule of thumb to build the references and drive the model to learn. Then, the best result was found, and its parameter setting was used as the final choice.

**Double Thresholds and Amplification Factor in Edge-preserving Smoothness Loss.** The double thresholds (α and β) in the edge-preserving smoothness loss directly determine the final position of the detected edge. It is easy to lose details when the thresholds are too large, and a large amount of noise will be retained when they are too small. The amplification factor (γ) changes the amplitude of the gradient at the edge, and an inappropriate release may trigger additional color variations. Considering that a great change in any color channel can indicate the presence of an edge, alternative parameters were set based on the theoretical maximum of the single-channel gradient amplitude. The larger (β) of the double thresholds was taken as 0.304, 0.707, 1.414 and 2.828, which were halved to obtain the smaller threshold (α), combined with γ = 1, 2, 3 for comparison. The results are shown in Table 3. As shown in the table, both metrics at γ=1 were poor. Although the SSIM was similar, the PSNR was better when γ=2 compared to γ=3 with the same β. And, the PSNR achieved the highest value at β=1.414. Thus, α=0.707, β=1.414 and γ=2 were chosen as the double thresholds and the amplification factor in the edge-preserving smoothness loss. These values may build good references for most low-light images.

#### 4.2.2. Effect of Inverse Transformation

The main purpose of the inverse transformation is to reduce the nonlinearity of the subsequent parameter extraction process and to facilitate the use of a simple model to obtain excellent performance. Under the same PGN structure, the performance of the two schemes in extracting directly from the original low-light image (namely, “without inverse”) and from the inverse transformation result (namely, “with inverse”) is shown in Table 4. It can be seen that the inverse transformation led to improvements of 0.04 in the SSIM and 1.00 in the PSNR. The scheme of first inverting and then extracting obtained better performance. This shows that the inverse transformation is conducive to the simplification of the PGN.

#### 4.2.3. Effect of Loss Functions

**Figure 5:**
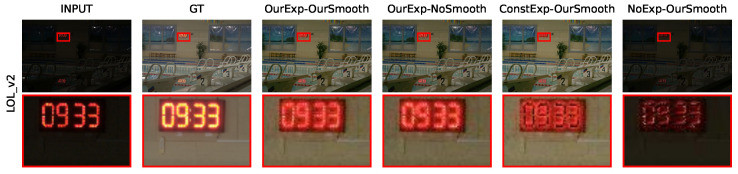
A comparison of the ablation of loss functions. The left side of “-” in each title indicates the state of brightness loss (Our: contrast-preserving brightness loss; Const: fixed-target-exposure loss; No: not use), and the right side indicates the state of smoothness loss.

Contrast-preserving brightness loss and edge-preserving smoothness loss realize the extraction and retention of the original brightness information and edge information separately. A comparison was carried out by using the fixed-target-exposure loss (approximately equivalent to the contrast-preserving brightness loss with each element of $E_{\mathrm{exptar}}$ as 0.6) and discarding the corresponding loss function. The results are shown in Figure 5 and Table 5.

As can be seen in Figure 5, the scheme of fixed-target-exposure loss plus edge-preserving smoothness loss showed a problem of abnormal color distribution. The numbers on the digital clock appeared black, contrary to the expectation. And, the specific numbers were hardly visible before amplification. Discarding the brightness loss directly led to the whole image being black. It only achieved a weak background brightness enhancement, and the original clock part with high brightness was suppressed. As a result of discarding the smoothness loss, the wall tiles under the digital clock appeared slightly more noisy. The edges of the digital parts were also more vague and had larger halos. The quantitative comparison in Table 5 also shows that the model output under our loss is closest to the expected target.

### 4.3. Experimental Configuration

Experiments were conducted on commonly used LLIE public datasets to compare the performance of the ZRCRN with several other SOTA methods: two traditional methods (LECARM [12], SDD [38]), two supervised learning methods (StableLLVE [39], URetinex_Net [40]) and three unsupervised learning methods (SCI [41], EnlightenGAN [17], Zero_DCE [18]). Each was implemented using the public code. The traditional methods ran on a CPU (Intel (R) Core (TM) i7-10870H). The learning-based methods, including our ZRCRN, loaded the specified checkpoints and ran on a GPU (NVIDIA RTX A6000). SCI had three available checkpoints, and the “difficult” version was selected here.

The experimental datasets include two full-reference (FR) datasets (LOL [42], LOL_v2 [36] (test part of real_captured)) and three no-reference (NR) datasets (DICM [43], LIME [13], MEF [44]). The performance of the nine algorithms was compared qualitatively and quantitatively. Then, the adaptability of these methods to advanced image tasks was evaluated by a night face detection experiment on the Dark Face [45] dataset.

### 4.4. Benchmark Evaluations

#### 4.4.1. Qualitative Comparison

**Figure 6:**
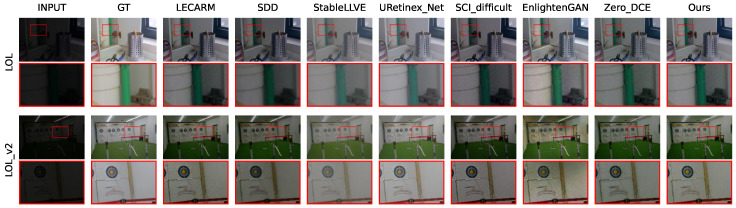
The qualitative comparison of eight methods on FR datasets with the input and ground truth (GT).

**Figure 7:**
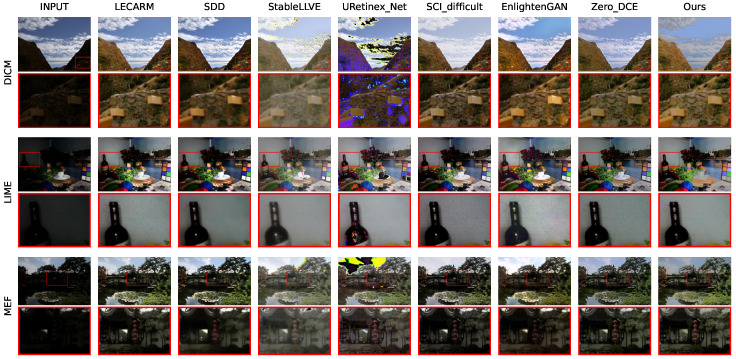
The qualitative comparison of eight methods on NR datasets with the input.

The qualitative comparisons of each method on each dataset are shown in Figure 6 and Figure 7, where the first row indicates the method to which each column belongs, and the first column indicates the dataset to which each row belongs. The parts in the red boxes are enlarged to facilitate comparison. It can be seen that each method could achieve a certain degree of enhancement. But, StableLLVE and URetinex_Net, using supervised learning strategies, showed significantly abnormal colors on the DICM and MEF datasets. Their application scenarios are severely limited. The enhancement results of the two traditional methods (LECARM and SDD) and the unsupervised method SCI on each dataset were somewhat dark. They could not achieve sufficient brightness improvement. Our ZRCRN resulted in good enhancement on all datasets and has the ability to suppress noise, which is most evident in the comparison on the LIME dataset. In the image of the LIME dataset enhanced by the ZRCRN, the wall on the right side of the bottle is very smooth, while the other three unsupervised methods retained relatively severe noise.

#### 4.4.2. Quantitative Comparison

The quality of the images enhanced by each method was evaluated using two metrics on each type of dataset separately. The SSIM [37] and PSNR were used for the FR datasets. BRISQUE [46] and NIQE [47] were used for the NR datasets. The comparisons are shown in Table 6 and Table 7. Two supervised learning methods achieved the top two mean SSIMs on LOL and LOL_v2, but their metrics on the three NR datasets ranked almost in the last two, which shows great application limitations. Four learning methods that do not require references from the datasets achieved the optimal four mean NIQE values on the NR datasets and also achieved excellent SSIMs and PSNRs on the FR datasets, which reflects their splendid generalization ability. Our ZRCRN obtained the best mean BRISQUE, and the other three metrics also ranked second among the four unsupervised methods.

In addition, the efficiency of each method was evaluated using FLOPs, the count of model parameters (#Params) and the average single-image processing time (denoted as TIME, in ms, calculated by dividing the processing time of all images in the test part of real_captured in LOL_v2 by the count of images contained in the same dataset). The results are shown in Table 8 (FLOPs and #Params are only calculated for learning-based methods). All of the metrics of our ZRCRN ranked first. The speed is more than twice that of Zero_DCE and EnlightenGAN with similar quality. Although the speed is not significantly improved compared to SCI, the accuracy is effectively improved by 0.07 for the mean SSIM and 1.77 for the mean PSNR on the LOL and LOL_v2 datasets.

### 4.5. Night Face Detection

**Figure 8:**
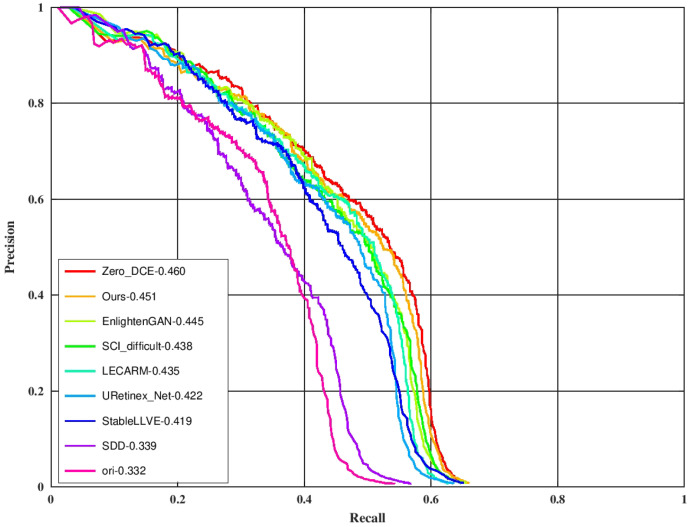
A comparison of enhancement methods on the DARK FACE dataset. “ori” indicates no enhancement. Targets were detected by DSFD from the outputs of each enhancement method to obtain the metric.

One of the main applications of high-speed enhancement methods is to extend the applicable scenarios for advanced image tasks such as target detection and target tracking. The improvement of the detection precision by each enhancement method was tested on the night face detection task. The Dual Shot Face Detector [48] (DSFD) with the official checkpoint (trained on the WIDER FACE dataset [49]) was used as the detector. One hundred low-light images with their localization labels were randomly selected from the training set of the DARK FACE dataset to form the testing set. The original low-light input image was first processed by each enhancement method and then underwent target detection. The evaluation tool (https://github.com/Ir1d/DARKFACE_eval_tools (accessed on 10 May 2024)) was used to draw the AP results at the IoU threshold of 0.5, and the results are shown in Figure 8 (label the scheme in which images are detected directly without enhancement as “ori”). As can be seen from the figure, the LLIE method can effectively improve the detection performance, and the effect of ours is similar to ZERO_DCE and is better than other methods. The total processing time is equivalent to the sum of the processing time of each enhancement method and the same DSFD detection time. Our ZRCRN is still the fastest.

## 5. Discussion

The ZRCRN proposed in this paper establishes a double-layer convolutional neural network based on the CRM to extract exposure ratio parameters to achieve LLIE. Combined with the two new loss functions, the ZRCRN can effectively improve efficiency without requiring references from the dataset. The sigmoid CRF and the corresponding CRM can be used in the learning models directly. Inversely transforming the input low-light image back to the incident radiation map helps to obtain high-quality enhancement with a more streamlined model. The contrast-preserving brightness loss and edge-preserving smoothness loss can also be used to constrain the training of other enhancement models. Appropriate enhancements of the input image features in the loss function combined with the excellent fitting ability of the learning models may bring about effective performance improvements. In addition, with the advantage of high efficiency, the ZRCRN might be an appropriate pre-processing method in object detection. But, the precision is still not very good. More diverse training data could help. Further work will be carried out to address this issue.

## 6. Conclusions

In this paper, we propose a high-speed zero-reference LLIE method, namely, the ZRCRN. The ZRCRN is established based on the CRM to achieve enhancement. High-quality outputs can be obtained with only one transformation using the BTF. The inverse CRF transformation reduces the need for a complex model structure in the extraction of enhancement parameters. Both can effectively improve efficiency. Contrast-preserving brightness loss and edge-preserving smoothness loss are designed to constrain the training. They do not need references from datasets and directly generate the expected targets from the features of low-light images, which can further retain the key information in the original input and is conducive to improving the quality of the enhancement. The experimental results on several LLIE datasets and the DARK FACE face detection dataset have revealed the advantages of our method when compared with several state-of-the-art methods. More work will be carried out to further improve precision and explore its application in object detection. Our source code is available on the project website.

## Figures and Tables

**Table 1:** Comparison for parameter extraction network.

	SSIM	PSNR	TIME/ms
**m1**	0.44	12.32	**0.41**
**m2-n1**	0.69	17.99	0.50
**m2-n2**	**0.69**	**18.20**	*0.51*
**m2-n4**	*0.68*	*17.74*	0.61
**m3-n1**	0.54	15.80	0.56
**m3-n2**	0.61	16.98	0.59
**m3-n4**	0.61	16.98	0.73
**m4-n1**	0.59	17.28	0.64
**m4-n2**	0.60	17.48	0.64
**m4-n4**	0.68	17.67	0.87

**Table 2:** Comparison for contrast-preserving brightness loss.

	SSIM	PSNR	SSIM-EXPTAR2BONL
**a = 0.0**	0.67	17.13	0.4
**a = 0.1**	*0.69*	17.73	0.65
**a = 0.2**	**0.69**	**18.20**	**0.73**
**a = 0.3**	0.69	18.09	0.73
**a = 0.4**	0.68	*17.84*	*0.69*
**a = 0.5**	0.64	16.94	0.65

**Table 3:** Comparison for edge-preserving smoothness loss.

	SSIM	PSNR
γ=1, β=0.304	0.69	17.78
γ=1, β=0.707	0.68	17.33
γ=1, β=1.414	0.67	16.85
γ=1, β=2.828	0.43	11.67
γ=2, β=0.304	0.69	17.98
γ=2, β=0.707	0.69	18.14
γ=2, β=1.414	**0.69**	**18.20**
γ=2, β=2.828	*0.69*	18.01
γ=3, β=0.304	0.69	17.85
γ=3, β=0.707	0.69	17.94
γ=3, β=1.414	0.69	*18.05*
γ=3, β=2.828	0.69	17.87

**Table 4:** Results of ablation of inverse transformation.

	SSIM	PSNR
**With inverse**	**0.69**	**18.20**
**Without inverse**	0.65	17.2

**Table 5:** Results of ablation of loss functions.

	SSIM	PSNR
**OurExp-OurSmooth**	**0.69**	**18.20**
**OurExp-NoSmooth**	0.69	18.00
**ConstExp-OurSmooth**	*0.68*	*17.93*
**NoExp-OurSmooth**	0.28	10.02

**Table 6:** Quantitative comparison for quality on two FR datasets.

	LOL		LOL_v2		Mean	
	SSIM	PSNR	SSIM	PSNR	SSIM	PSNR
**LECARM**	0.67	14.42	0.67	17.66	0.67	16.04
**SDD**	0.67	13.25	0.7	16.51	0.69	14.88
**StableLLVE**	0.78	*17.25*	0.78	19.8	0.78	18.52
**URetinex_Net**	**0.86**	**19.55**	**0.85**	**20.66**	**0.85**	**20.11**
**SCI**	0.63	13.8	0.63	17.3	0.63	15.55
**EnlightenGAN**	*0.74*	17.44	*0.74*	*18.62*	*0.74*	*18.03*
**Zero_DCE**	0.7	14.84	0.69	18.13	0.69	16.49
**Ours**	0.72	16.44	0.69	18.2	0.71	17.32

**Table 7:** Quantitative comparison for quality on three NR datasets.

	DICM		LIME		MEF		Mean	
	BRISQUE	NIQE	BRISQUE	NIQE	BRISQUE	NIQE	BRISQUE	NIQE
**LECARM**	26.70	4.24	*20.97*	3.99	*19.74*	**3.00**	22.47	3.74
**SDD**	30.74	3.94	27.09	4.01	29.73	3.86	29.19	3.94
**StableLLVE**	33.73	4.24	29.12	4.20	34.11	4.30	32.32	4.25
**URetinex_Net**	28.04	4.07	26.38	4.41	25.14	3.73	26.52	4.07
**SCI**	23.04	3.67	20.68	4.17	19.24	3.16	20.99	3.67
**EnlightenGAN**	*23.80*	3.50	**19.62**	**3.54**	21.06	*3.07*	*21.49*	**3.37**
**Zero_DCE**	25.04	*3.57*	22.17	*3.90*	21.09	3.03	22.77	*3.50*
**Ours**	**21.83**	**3.40**	22.39	3.87	**18.25**	3.23	**20.82**	3.50

**Table 8:** Quantitative comparison for efficiency.

	FLOPs/G	#Params/k	TIME/ms
**LECARM**	-	-	71.310
**SDD**	-	-	5952.140
**StableLLVE**	165.316	4316.259	3.306
**URetinex_Net**	939.912	340.105	89.750
**SCI**	0.272	0.258	0.676
**EnlightenGAN**	275.020	8636.675	19.390
**Zero_DCE**	*85.769*	*79.416*	*1.300*
**Ours**	**0.081**	**0.075**	**0.518**

## Data Availability

The corresponding code is openly available at https://github.com/kazei7/ZRCRN (accessed on 10 May 2024). The LOL, LOL_v2, DICM, LIME and MEF datasets used in the research are available from the website https://github.com/Li-Chongyi/Lighting-the-Darkness-in-the-Deep-Learning-Era-Open (accessed on 10 May 2024). The DARK FACE dataset used in the research is available from the website https://flyywh.github.io/CVPRW2019LowLight/ (accessed on 10 May 2024).
